# Extent of Surgery and Survival of Osteosarcoma: A Retrospective Population-Based Study

**DOI:** 10.7759/cureus.56030

**Published:** 2024-03-12

**Authors:** Connor J Tupper, Emily A Reeson, Michael R Burdyny, Vincent P Eaton, Peter T Silberstein

**Affiliations:** 1 Department of Orthopaedics, Creighton University School of Medicine, St. Joseph’s Hospital and Medical Center, Phoenix, USA; 2 Department of Orthopaedics, Creighton University School of Medicine, Omaha, USA; 3 Department of Hematology and Oncology, Creighton University School of Medicine, Omaha, USA

**Keywords:** adjuvant chemotherapy, neoadjuvant chemotherapy, cancer survival, cancer registry, limb-sparing surgery, extent of surgery, bone tumor, osteosarcoma

## Abstract

Background

Osteosarcoma (OSC) is the most common primary bone tumor and is often managed surgically. Few prior investigations have assessed differences in OSC survival by specific surgical techniques at a national registry level. We sought to compare survival based on surgical subtypes for OSC patients in the Surveillance, Epidemiology, and End Results (SEER) database.

Methodology

We searched the SEER database for malignant OSCs diagnosed between 2000 and 2019 which were surgically managed. Separate survival comparisons were made for one and five years for wide excision (local tumor destruction or resection versus partial resection) and radical excision (radical resection with limb-sparing versus limb amputation with or without girdle resection).

Results

A total of 4,303 patients were included, of whom 3,587 were surgically managed. There were no survival differences between local destruction and partial resection (hazard ratio = 0.826, p = 0.303). However, younger age, lower staging, and management without radiation were associated with improved survival. The radical excision comparison showed limb amputation was associated with worse survival than limb-sparing surgery (hazard ratio = 1.531, p < 0.001). Younger age, female sex, lower stage, receipt of chemotherapy, and neoadjuvant plus adjuvant chemotherapy were associated with improved survival while Black and American Indian or Alaska Native were associated with worse survival.

Conclusions

Our findings show that patients managed with limb-sparing radical resection survived significantly compared to limb amputation. There were no differences in survival for wide excision surgeries. The use of a combination of neoadjuvant and adjuvant chemotherapy also yields improved survival. OSC survival may be optimized with limb-sparing surgery with a combination of neoadjuvant and adjuvant chemotherapy.

## Introduction

Osteosarcoma (OSC) is the most common primary malignant bone tumor [1]. The tumor is primarily diagnosed in the pediatric population and arises insidiously with patients often presenting with pain or dysfunction in the affected area, most commonly the metaphysis of the long bones in the lower extremities [2]. A growing number of treatment options exist for the management of OSC [2]. However, the mainstay of OSC management is immediate surgical intervention or neoadjuvant systemic chemotherapy (high-dose methotrexate with leucovorin rescue, doxorubicin, cisplatin, ifosfamide, and/or etoposide) followed by definitive surgical excision [3,4].

Surgical management alone was the primary treatment for OSC before neoadjuvant chemotherapy became widely used [3]. Surgical resection often results in wide surgical margins with the focus on saving the patient’s life, and less so on salvaging their physical functioning [3]. Neoadjuvant therapy, however, improves survival as well as destroys the micrometastasis that may be missed on gross sectioning, thus allowing for margins that conserve more bone and more functional capacity consequently [5]. While some investigations have compared outcomes based on varying surgical invasiveness [6], no study has confirmed differences in outcomes based on the extent of surgery (i.e., wide or radical surgical excision) at the level of a large national cancer registry.

Further exploration of surgical outcomes is needed to guide physician decision-making in the midst of numerous and growing numbers of OSC treatment options. Thus, the objective of this study was to compare survival after OSC diagnosis based on the extent of surgery for OSC patients in the Surveillance, Epidemiology, and End Results (SEER) database.

The preliminary results of this project were previously presented as a visual abstract at the 2023 Medical Student Orthopedic Society Research Symposium.

## Materials and methods

This study was approved by the Creighton University Institutional Review Board. The SEER-18 database was used to identify malignant OSC cases diagnosed between 2000 and 2019 with site codes 9180-9186 and 9192-9194. SEER patient data is unidentified so there is minimal risk for patient confidentiality breaches. Patients were excluded if the cause of death was not associated with their cancer, according to the SEER cancer-specific death categorization. Cancer-specific survival (CSS) was traced up to 60 months after diagnosis. Variables selected included age, sex, race, stage at diagnosis, tumor primary site, surgical status, extent of surgery, radiation status, chemotherapy status, time from diagnosis to treatment, and timing between systemic therapy and surgery.

Age was split into three groups to account for the significant burden in the pediatric population: 0-14 years, 15-29 years, and 30+ years. Sex included males and females. Race included non-Hispanic White, non-Hispanic Black, Hispanic, Asian or Pacific Islander, and American Indian or Alaska Native. The stage at diagnosis was based on the SEER-based combined staging resulting in the designation of localized, regional, or distant. The tumor’s primary site was classified as axial or appendicular. Axial classification included the face and skull, mandible, vertebral column, thorax, pelvis, sacrum, and coccyx. Appendicular classification included the upper limbs, scapula, and lower limbs. Surgical status indicated whether or not the patient received surgery.

The extent of surgical resection was used to classify surgical type as wide or radical excision. Wide excision included cases categorized as local tumor destruction or resection, or partial resection involving resection of the tumor and surrounding normal tissue. Radical excision included cases categorized as radical excision with limb-sparing, and limb amputation or major amputation involving part of the shoulder or pelvic girdle. Radiation and chemotherapy status indicated whether or not the patient received radiation or chemotherapy, respectively. If the radiation and chemotherapy status was unknown, the patient was grouped in the “no” designation, based on the SEER coding system. Time from diagnosis to treatment was measured in months and was divided into three groups: within one month, between one to two months, and over two months. The timing between systemic therapy and surgery was classified as neoadjuvant, adjuvant, or both neoadjuvant and adjuvant.

Kaplan-Meier curves were created to compare CSS within variables. Chi-square log-rank tests were performed to compare one- and five-year survival rates between surgical groups. Survival comparisons were made between those who did and did not receive surgery as well as surgeries of similar invasiveness, including a low-invasiveness and a high-invasiveness grouping. Cox proportional hazards regression analyses were completed to assess the effect of the aforementioned variables on CSS. Cox proportional hazard models assume the risk of the event of interest, which in this study is death, is independent and proportional between groups throughout follow-up, which can be assumed as cases are not linked. Chemotherapy status and order of surgery and systemic therapy were not included in the Cox regression for the low-invasiveness surgery analysis as the use of systemic therapy in this group was minimal. Patient cases without complete data on all variables included in the specific test were excluded from that analysis to minimize the risk of errors. The significance for all analyses was set at p < 0.05. SPSS version 28 (IBM Corp., Armonk, NY, USA) was used for all statistical analyses.

## Results

A total of 4,303 patients were collected. A total of 3,587 (83.4%) patients underwent surgical intervention, including 85 patients whose type of surgery was not defined and were therefore not included in the extent of surgery subanalyses. Of the 534 (14.9%) patients who underwent low-invasiveness surgery, 305 (57.1%) had local tumor destruction or excision, and 229 (42.9%) had partial excision. Of the 2,968 (82.7%) patients who underwent high-invasiveness surgery, 2,168 (73.0%) received radical excision with limb-sparing and 800 (27.0%) received limb amputation with or without girdle resection. The majority of patients (n = 3,399, 79.0%) underwent chemotherapy while 904 (21.0%) did not or their status was unknown. Of recorded cases, 620 (30.6%) patients received neoadjuvant systemic therapy, 335 (16.5%) received adjuvant systemic therapy, and 1,073 (52.9%) received both neoadjuvant and adjuvant systemic therapy. Of recorded cases, a total of 1,861 (46.5%) patients received treatment within one month of diagnosis, 1,590 (39.7%) between one and two months, and 554 (13.8%) over two months after diagnosis. Complete descriptive statistics are included in Table 1.

**Table 1:** Patient demographics and Pearson chi-square results. Statistical significance at p < 0.05.

Variables	All patients, N (%)	Alive at one year, N (%)	Deceased at one year, N (%)	P-value	Alive at five years, N (%)	Deceased at five years, N (%)	P-value
Age				<0.001			<0.001
0–14 years	1,332 (31.0)	1,272 (32.9)	60 (13.9)		996 (34.5)	336 (23.7)	
15–29 years	1,567 (36.4)	1,480 (38.2)	87 (20.2)		1,108 (38.4)	459 (36.4)	
30+ years	1,404 (32.6)	1,120 (28.9)	284 (65.9)		782 (27.1)	622 (32.6)	
Sex				0.187			<0.001
Male	2,377 (55.2)	2,126 (54.9)	251 (58.2)		1,518 (52.6)	859 (60.6)	
Female	1,926 (44.8)	1,746 (45.1)	180 (41.8)		1,368 (47.4)	558 (39.4)	
Race				0.561			0.260
Non-Hispanic White	2,100 (49.0)	1,876 (48.7)	224 (52.0)		1,395 (48.6)	705 (49.8)	
Non-Hispanic Black	1,161 (27.1)	567 (14.7)	62 (14.4)		410 (14.3)	219 (15.5)	
Hispanic	629 (14.7)	1,055 (27.4)	106 (24.6)		794 (27.7)	367 (25.9)	
Asian or Pacific Islander	362 (8.4)	328 (8.5)	34 (7.9)		252 (8.8)	110 (7.8)	
American Indian or Alaska Native	34 (0.8)	29 (0.8)	5 (1.2)		19 (0.7)	15 (1.1)	
Stage at diagnosis				<0.001			<0.001
Localized	1,244 (37.8)	1,207 (40.4)	37 (12.3)		1,035 (46.1)	209 (20.1)	
Regional	1,281 (39.0)	1,231 (41.2)	50 (16.6)		931 (41.4)	350 (33.7)	
Distant	762 (23.2)	547 (18.3)	215 (71.2)		281 (12.5)	481 (46.3)	
Tumor primary site				<0.001			<0.001
Appendicular	3,305 (78.0)	3,072 (80.2)	233 (57.2)		2,344 (82.1)	961 (69.6)	
Axial	932 (22.0)	758 (19.8)	174 (42.8)		512 (17.9)	420 (30.4)	
Radiation therapy status				<0.001			<0.001
No radiation	3,912 (90.9)	3,580 (92.5)	332 (77.0)		2,732 (94.7)	1,180 (83.3)	
Received radiation	391 (9.1)	292 (7.5)	99 (23.0)		154 (5.3)	237 (16.7)	
Systemic therapy status				<0.001			0.917
No systemic therapy	904 (21.0)	729 (18.8)	175 (40.6)		605 (21.0)	299 (21.1)	
Received systemic therapy	3,399 (79.0)	3,143 (81.2)	256 (59.4)		2,281 (79.0)	1,118 (78.9)	
Surgery to systemic therapy sequence				<0.001			<0.001
Neoadjuvant	620 (30.6)	592 (30.4)	28 (35.4)		440 (29.6)	180 (33.3)	
Adjuvant	335 (16.5)	306 (15.7)	29 (36.7)		225 (15.1)	11 (20.4)	
Neoadjuvant and adjuvant	1,073 (52.9)	1,051 (53.9)	22 (27.8)		823 (55.3)	250 (46.3)	
Time from diagnosis to treatment				0.815			0.508
<1 month	1,861 (46.5)	1,703 (46.5)	158 (45.8)		1,278 (46.9)	583 (45.5)	
1–2 months	1,590 (39.7)	1,448 (39.6)	142 (41.2)		1,079 (39.6)	511 (39.9)	
2+ months	554 (13.8)	509 (13.9)	45 (13.0)		366 (13.4)	188 (14.7)	
Extent of surgery: wide excision				0.031			0.816
Local tumor destruction or excision	305 (57.1)	275 (55.8)	30 (73.2)		221 (56.8)	84 (57.9)	
Partial resection	229 (42.9)	218 (44.2)	11 (26.8)		168 (43.2)	61 (42.1)	
Extent of surgery: radical excision				<0.001			<0.001
Radical excision with limb-sparing	2,168 (73.0)	2,096 (74.0)	72 (53.3)		1,653 (76.6)	515 (63.6)	
Major amputation	800 (27.0)	737 (26.0)	63 (46.7)		505 (23.4)	295 (36.4)	

**Figure 1:**
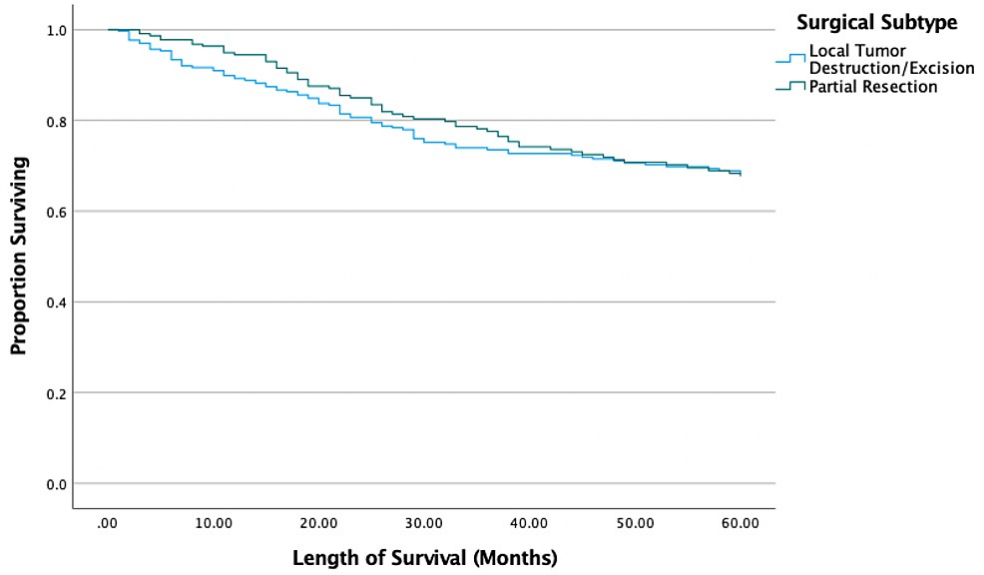
Kaplan-Meier results comparing wide excision.

**Figure 2:**
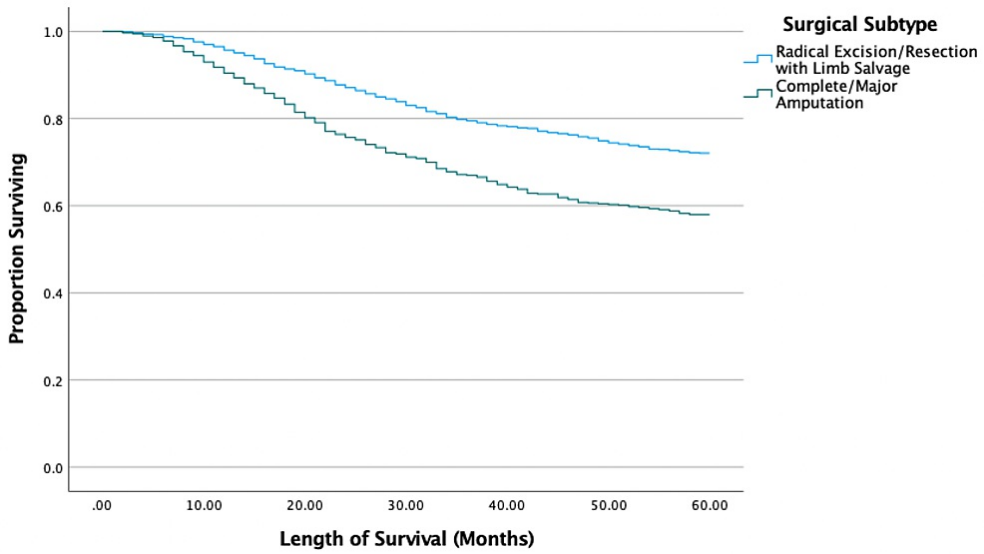
Kaplan-Meier results for radical excision.

**Figure 3:**
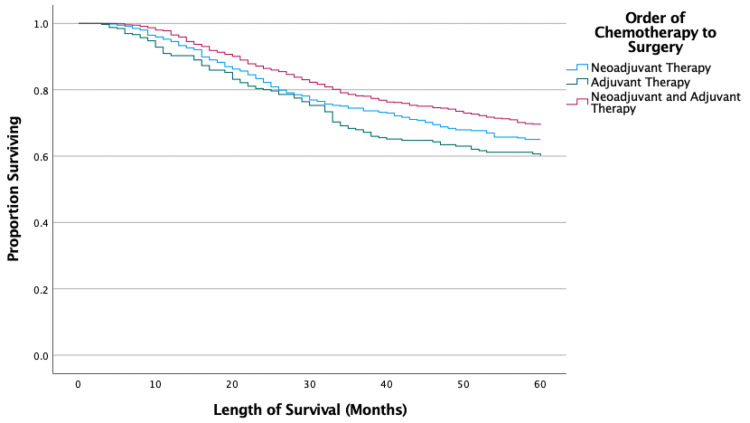
Kaplan-Meier results for sequence of chemotherapy relative to surgery.

Kaplan-Meier results showed surgical patients had significantly longer survival than the nonsurgical group (p < 0.001). The type of wide excision (local tumor destruction or resection versus partial resection) did not significantly affect survival (p = 0.845) (Figure 1). However, the type of radical excision did affect survival as longer survival was seen with radical resection with limb-sparing compared to limb amputation with or without girdle resection (p < 0.001) (Figure 2). Patients who received chemotherapy had significantly improved survival compared to those who did not (p = 0.002). The sequence of chemotherapy and surgery showed no differences between neoadjuvant and adjuvant chemotherapeutic timing (p = 0.192), but those who underwent both neoadjuvant and adjuvant chemotherapy had significantly improved survival compared to neoadjuvant-only (p = 0.024) and adjuvant-only (p < 0.001) groups (Figure 3).

Chi-square analysis indicated age, stage, primary site, wide excision, radical excision, radiation therapy status, chemotherapy status, and timing between surgery and systemic therapy are each significantly associated with one-year survival. No relationships were present between one-year survival and sex, race, and time to treatment from diagnosis. Age, sex, stage, primary site, radical excision, radiation therapy status, and order of surgery and systemic therapy are significantly associated with five-year survival. No relationships were present between five-year survival and race, wide excision, chemotherapy status, and time to treatment from diagnosis. Complete results are seen in Table 1.

Cox regression analysis for the wide excision comparison between local tumor destruction or resection and partial resection showed age, stage, and radiation status all significantly influenced survival. Compared to the 0-14-year-old group, the 30+-year-old group did not survive as long (p < 0.001, hazard ratio (HR) = 2.498, 95% confidence interval (CI) = 1.455-4.290). Compared to the localized stage, patients with regional (p = 0.004, HR = 1.865, 95% CI = 1.216-2.862) or distant staging (p < 0.001, HR = 8.902, 95% CI = 5.465-14.499) survived shorter. Those who received radiation had significantly shorter survival compared to those who were managed without radiation therapy (p = 0.001, HR = 2.133, 95% CI = 1.349-3.373). No other significant findings were found for sex, race, primary site, type of surgery, and time to treatment in this assessment. Complete results are shown in Table 2.

**Table 2:** Cox proportional hazards model results of wide excision. Statistical significance at p < 0.05.

Variables	P-value	Hazard ratio	95% confidence interval
Age (Standard: 0–14 years)	-	-	-
15–29 years	0.272	1.368	0.782-2.390
30+ years	<0.001	2.498	1.455-4.290
Sex (Standard: male)	-	-	-
Female	0.880	0.972	0.675-1.401
Race (Standard: non-Hispanic White)	-	-	-
Non-Hispanic Black	0.729	1.094	0.659-1.815
Hispanic	0.798	1.057	0.690-1.621
Asian or Pacific Islander	0.282	0.667	0.319-1.394
American Indian or Alaska Native	0.438	2.217	0.297-16.556
Stage (Standard: localized)	-	-	-
Regional	0.004	1.865	1.216-2.862
Distant	<0.001	8.902	5.465-14.499
Primary site (Standard: appendicular)	-	-	-
Axial	0.307	0.794	0.509-1.236
Radiation therapy (Standard: no radiation)	-	-	-
Received radiation	0.001	2.133	1.349-3.373
Wide excision surgery (Standard: local tumor destruction or excision)	-	-	-
Partial resection	0.303	0.826	0.574-1.188
Time to treatment (Standard: <1 month)	-	-	-
1–2 months	0.696	0.925	0.624-1.370
2+ months	0.543	0.844	0.489-1.457

Cox regression analysis for the radical excision comparison between radical resection with limb-sparing and limb amputation with or without girdle resection showed age, sex, race, stage at diagnosis, surgical type, chemotherapy status, and order of surgery and systemic therapy influenced survival. Both the 15-29-year-old group (p = 0.011, HR = 1.301, 95% CI = 1.062-1.595) and 30+-year-old group (p < 0.001, HR = 2.011, 95% CI = 1.540-2.627) did not survive as long as the 0-14-year-old group. Female patients had greater survival (p = 0.043, HR = 0.832, 95% CI = 0.696-0.995) than males. Compared to non-Hispanic White patients, non-Hispanic Black (p = 0.016, HR = 1.351, 95% CI = 1.058-1.724) and American Indian or Native Alaskan (p < 0.001, HR = 3.546, 95% CI = 1.714-7.336) survived shorter. No differences were observed between the survival of non-Hispanic White patients and Hispanic and Asian or Pacific Islander patients. Compared to patients with localized staging at diagnosis, both regional staging (p = 0.002, HR = 1.424, 95% CI = 1.139-1.780) and distant staging (p < 0.001, HR = 3.982, 95% CI = 3.141-5.049) had shorter survival. The type of surgical intervention played a significant role, as those who underwent limb amputation with or without girdle resection survived shorter compared to those who underwent radical excision with limb-sparing (p < 0.001, HR = 1.531, 95% CI = 1.275-1.838). Chemotherapy treatment was associated with improved survival compared to those who did not undergo chemotherapy (p = 0.010, HR = 0.070, 95% CI = 0.009-0.532). Compared to neoadjuvant therapy, those who underwent both neoadjuvant therapy and adjuvant therapy had improved survival (p = 0.042, HR = 0.819, 95% CI = 0.676-0.993). No differences were seen between patients receiving neoadjuvant chemotherapy and patients receiving adjuvant chemotherapy. No other significant findings were found for the primary site, radiation status, and time to treatment. Complete results are shown in Table 3.

**Table 3:** Cox proportional hazards model results of radical excision. Statistical significance at p < 0.05.

Variables	P-value	Hazard ratio	95% confidence interval
Age (Standard: 0–14 years)	-	-	-
15–29 years	0.011	1.301	1.062-1.595
30+ years	<0.001	2.011	1.540-2.627
Sex (Standard: male)	-	-	-
Female	0.043	0.832	0.696-0.995
Race (Standard: non-Hispanic White)	-	-	-
Non-Hispanic Black	0.016	1.351	1.058-1.724
Hispanic	0.639	1.052	0.852-1.298
Asian or Pacific Islander	0.317	1.167	0.862-1.581
American Indian or Alaska Native	<0.001	3.546	1.714-7.336
Stage (Standard: localized)	-	-	-
Regional	0.002	1.424	1.139-1.780
Distant	<0.001	3.982	3.141-5.049
Primary site (Standard: appendicular)	-	-	-
Axial	0.074	1.289	0.976-1.702
Radical excision surgery (Standard: radical excision with limb-sparing)	-	-	-
Major Amputation	<0.001	1.531	1.275-1.838
Chemotherapy status (Standard: no)	-	-	-
Received chemotherapy	0.010	0.070	0.009-0.532
Chemotherapy sequence (Standard: neoadjuvant)	-	-	-
Adjuvant	0.390	0.881	0.660-1.176
Neoadjuvant and adjuvant	0.042	0.819	0.676-0.993
Radiation therapy (Standard: no radiation)	-	-	-
Received Radiation	0.075	1.408	0.966-2.052
Time to treatment (Standard: <1 month)	-	-	-
1–2 months	0.615	0.953	0.791-1.148
2+ months	0.285	0.846	0.623-1.149

## Discussion

This retrospective analysis of 4,303 patients in the SEER database indicates surgical type influences the survival of OSC patients who receive radical surgical resection. Similar to prior reports, OSC patients who underwent radical excision with limb-sparing had improved survival compared to those who underwent limb amputation with or without girdle resection [7,8]. This finding is consistent with the preferred management strategy in clinical practice. Despite higher local recurrence rates compared to amputation, the five-year survival rates are similar to those between limb-sparing surgery and amputation [9]. In addition to similar survival, limb-sparing surgery is also more commonly used in clinical practice (given adequate margins can be achieved) due to improved postoperative functioning, cosmetic satisfaction, and overall cost reduction [9-11]. In concordance with these advantages, the results of this study confirm the current clinical practice to favor limb-sparing surgery over amputation.

In addition, combined adjuvant and neoadjuvant chemotherapy was found to improve survival compared to chemotherapy at either timeframe alone. Previous studies have shown mixed results and subsequent recommendations regarding the use of adjuvant and neoadjuvant chemotherapy to manage OSC [12-17]. Differences in results between these trials may be attributed to the variable response of each case to chemotherapeutic agents, which can be influenced by tumor histology and histologic response to chemotherapy [4,14,18-20]. The Pediatric Oncology Group Study POG-8651, a key trial comparing adjuvant to neoadjuvant therapy, showed similar five-year survival between the two groups [21]. Fewer investigations have assessed combined neoadjuvant and adjuvant chemotherapy compared to monotherapy. The results of this study favor the administration of chemotherapy in combination before and after surgery, which is agreeable with other large cancer database studies [8]. However, because of the high side effect and toxicity profile of the agents used, a thorough evaluation should be completed to take into consideration all these factors before initiating a therapeutic plan for OSC patients.

Analysis of survival based on demographic variables in this study reflects much of what is already established in OSC literature. Similar to prior large database studies, this study found the 0-14-year-old group had the best survival relative to older groups [7,22-24]. Differences in survival by sex also aligned with prior reports, as females experienced improved survival relative to males [7,22]. Additionally, similar to Cole et al., American Indians or Alaska Natives survived significantly shorter than non-Hispanic Whites [22]. Unlike other studies in the SEER database and National Cancer Database (NCDB), this study also showed that non-Hispanic Black patients survived significantly shorter than non-Hispanic Whites [7,22]. Despite similarities in the patient populations, the SEER study differences may be attributed to variance in the timeframe of data collected, while the NCDB study differences may be attributed to the different variables used for the Cox regression analyses.

The results of this study further confirmed the clinical characteristics of OSC that have been investigated in prior studies. Advanced staging is a well-known risk factor for poor prognosis in OSC patients [7,22,25-27]. Surgical intervention is the mainstay of OSC management to improve survival [23,25,26]. In large cancer database analyses, the treatment variable is most often dichotomous, including surgery and no surgery, thus leaving the specific type of surgery performed seldom reported [7,28]. Unlike prior analyses, however, this study did break out surgical type to investigate the largely unaddressed question of how surgical type affects survival on a national cancer database level. For the wide excision comparison, there were no significant differences observed between the groups at any time point. Such similar results will likely defer to surgeon preference for the type of surgical intervention when wide excision surgeries are planned, assuming adequate margins can be obtained. For the radical excision comparison, significant differences were observed favoring the radical excision with limb-sparing. This finding is similar to two investigations of OSC in the NCDB [7,28].

Finally, in the wide excision comparison, patients who received radiation therapy had significantly shorter survival relative to those who did not. This finding has been shown to be an independent risk factor for worse survival in other studies [23,27]. Reasons for shorter survival are likely related to the poor response of OSC to radiation as well as radiation more often utilized in the postoperative period, possibly related to inoperability, tumor advancement, or tumor recurrence [23,27].

Variables not found to be associated with improved survival on Cox regression included primary site and time to treatment. For the primary site, multiple cancer registry studies have suggested that lower extremity short bones are associated with improved survival compared to upper extremity short bones, and the pelvis or axial distribution is associated with worse survival [7,22,24,29]. Kaplan-Meier and log-rank findings in this study support these results, as patients with appendicular tumors had greater CSS compared to axial tumors. However, these results were not maintained on Cox regression. The pelvic primary site has especially poor survival. This is likely attributed to lower rates of surgical treatment likely due to larger tumor size, higher rates of metastasis, close regional involvement of other bony structures in the sacrum, older age at diagnosis, and longer duration between diagnosis and treatment at a specialized center [25]. Of these, this study did show that older age is associated with worse survival compared to younger patients; however, the time from diagnosis to treatment was not supported. While atypical for most malignancies, the findings in this study as well as others suggest time to treatment has little to no effect on survival in the short term [18]. The time of referral, establishing care, and beginning treatment at high-volume centers is likely the reason for similar survival rates seen between immediate intervention and potentially months before starting treatment [30]. Unless necessary for patient optimization, initiation of treatment should seldom be delayed. Nonetheless, this study does display that minor delays for any reason should not influence the prognosis of OSC patients.

This study is not without limitations. Despite having a large sample size from across the United States with the inclusion of multiple clinically important covariates, the retrospective nature of the study leads to conclusions being drawn from correlations and not from causational patterns as can be attained through prospective trials. Due to the large sample size comprising data from registries across the country, the generalizability of these findings is likely strong but may be less generalizable for groups with fewer patients such as ethnic minorities, or based on variables not included such as insurance status and treatment facility type. Also inherent to retrospective registry studies is the narrowing of a patient into a case made up of variables. This is an effective way to monitor overarching trends across a relatively large cohort, but with increasing breadth of analysis comes the loss of the intangible characteristics of individual patients that may help or hinder their biopsychosocial battle with their cancer diagnosis.

There are also variables not included in the analysis that may influence survival, such as OSC histology subtype, more specific primary site locations (e.g., upper versus lower extremity or long bone versus short bone), insurance status, surgical margins, or response to chemotherapy [7]. A potential bias of database studies is survival analyses that are not cancer-specific, such as those in NCDB. However, the utilization of SEER CSS coding for our analyses strengthens the credibility of our survival results. Further description of the extent of surgical resection may also facilitate appropriate comparisons between and within groups. While it is difficult to control for all factors that can influence survival in OSC patients, particularly with observational data from SEER which may pose concerns for bias, the multivariate analysis to control for the included covariates provides a strong platform to draw conclusions regarding the differences in CSS between surgical types for OSC patients.

## Conclusions

Surgical management is the primary mode of management for OSC, and this study has shown that limb-sparing radical excision is associated with improved survival compared to more extensive surgical management involving limb amputation. Our results also show similar survival between local and partial resections. The timing between surgery and systemic therapy, which is used in the majority of cases, influenced survival in high-invasive surgical cases as those who underwent neoadjuvant and adjuvant systemic therapy had greater survival compared to neoadjuvant alone. Our findings suggest surgical intervention with adequate margins, but sparing as much unaffected tissue as possible, can improve survival. Additionally, more aggressive systemic therapy involving treatment both before and after surgery may best improve survival. Future studies will benefit from implementing these findings in treatment plans and assessing additional variables that may influence the survival of OSC patients.
